# The identity of Alfred Wallace’s mysterious butterfly taxon *Lycaena nisa* solved: *Famegana nisa* comb. nov., a senior synonym of *F. alsulus* (Lepidoptera, Lycaenidae, Polyommatinae)

**DOI:** 10.3897/zookeys.966.51921

**Published:** 2020-09-09

**Authors:** Yu-Feng Hsu

**Affiliations:** 1 Department of Life Science, National Taiwan Normal University, Taipei, 116, Taiwan, ROC National Taiwan Normal University Taipei Taiwan

**Keywords:** *Famegana alsulus*, Formosa, lectotype designation, *Lycaena alsulus*, new synonymy, Taiwan, *Zizera taiwana*

## Abstract

*Lycaena nisa* Wallace, 1866 was described from Formosa (Taiwan) and is here recognized as a senior subjective synonym of *Lycaena alsulus* Herrich-Schäffer, 1869. It is resurrected to serve as the valid name, *Famegana nisa* (Wallace, 1866), **comb nov.** of the species commonly known as *Famegana alsulus*. The name *Zizera taiwana* Sonan, 1938 (**syn. nov.**), also described from Formosa, is recognized as a junior subjective synonym of *L. nisa*. Another name, *Zizeeria alsulus eggletoni* Corbet, 1941 (**syn. nov.**), described from Hong Kong is also considered a junior subjective synonym of *L. nisa*. Moreover, all former synonyms of *alsulus* automatically become new junior synonyms for *nisa*. This species occurs in the Oriental and Australian regions and western Pacific.

## Introduction

Anyone interested in natural history knows the name Alfred Russel Wallace, considered the father of zoogeography, who developed the idea of nature selection independently of Charles Darwin (Limolino et al. 2010; Beccaloni 2013). His achievements cover a variety of biological disciplines, including systematics. He described many plants and animals, mostly based on collections made by himself and his assistants. Nevertheless, he also worked on collections from other sources. A good example is a work on lepidopterous insects collected by Robert Swinhoe, an English biologist who worked as consul in Formosa (Taiwan) from 1860–1866. In collaboration with Frederic Moore, Wallace studied a collection made in Takaw [today’s Kaohsiung]. The butterfly portion of the collection was investigated by Wallace, the moth portion by Moore, and a joint paper was subsequently published as Wallace and Moore (1866). This paper has been regarded as the starting point of lepidopteran research of Taiwan (Shirôzu 1986). In this landmark work of the Lepidoptera of Taiwan, 46 diurnal species and 93 nocturnal species are mentioned. Wallace noted that most of the species in the collection were widespread species with distributions shared with India and Malay, but he recognized five species he considered distinctive and described them as new. These species were *Pontia niobe* Wallace, 1866, *Pieris formosana* Wallace, 1866, *Terias vagans* Wallace, 1866, *Euploea swinhoei* Wallace, 1866, and *Lycaena nisa* Wallace, 1866. The taxonomic status of the first four taxa have been clarified by various authors since. *Pontia niobe* is recognized as a subspecies of *Leptosia nina* (Fabricius, 1793) (Pieridae) (Yata 1985; Hsu et al. 2018). *Pieris formosana* is generally considered as either a subspecies of *Appias lyncida* Cramer, 1777 (Pieridae) (e.g., Shirôzu 1960) or a junior synonym of *A. lyncida eleonora* Boisduval, 1836 (Pieridae) (e.g., Yata 1985; Hsu et al. 2018). *Terias vagans* is recognized as a junior synonym of *Eurema laeta* (Boisduval, 1836) (Pieridae) by Yata (1989). *Euploea swinhoei* is considered a subspecies of *Euploea sylvester* (Fabricius, 1793) (Nymphalidae) (Ackery and Vane-Wright 1984; Morishita 1985).

**Figures 1–9.**
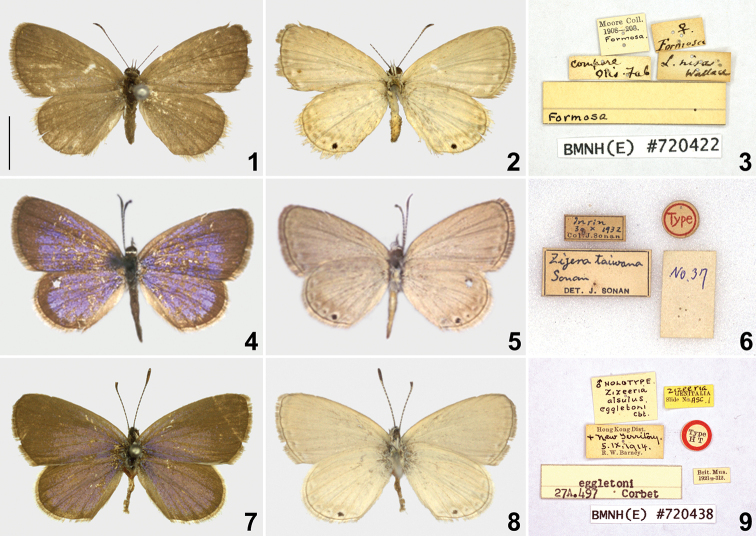
Type specimens of *Famegana nisa* (Wallace, 1866) **1–3** lectotype of *Lycaena nisa* Wallace, 1866 **4–6** holotype of *Lycaena taiwana* Sonan, 1938 **7–9** holotype of *Zizeeria alsulus eggletoni* Corbet, 1941. Scale bar: 0.5 cm.

The status of *Lycaena nisa*, however, remains ambiguous and has not been re-examined (Shirôzu 1986). Matsumura (1909) changed the generic assignment of *L. nisa* Wallace, 1866 to the genus *Zizera* but without giving any explanation. *Lycaena nisa* was excluded from a comprehensive checklist of Taiwan butterflies by Shirôzu (1960) and Shirôzu and Ueda (1992). During visits to the Natural History Museum, London (NHMUK) for a project on documenting information on the type specimens of Taiwan butterflies, the type material of *L. nisa* was retrieved from the Wallace collection. According to Wallace’s (1866) original description, a pair of syntypes were available for *L. nisa*, but only a female specimen (Figs 1–3) was successfully located in the museum. The features of the specimen fully conform to the description given by Wallace (Wallace and Moore 1866: 360–361). Interestingly, it also agrees with a species commonly known as *Famegana alsulus* (Herrich-Schäffer, 1869) in all aspects, and these two taxa are shown to be conspecific.

As *Lycaena nisa* was published three years prior to Herrich-Schäffer’s *Lycaena alsulus*, it takes the priority, and should be the valid name, invoking Article 23.1 of the ICZN (1999: 24). Although the name *alsulus* has been used for this lycaenid butterfly in the literature more than 25 times in the last 50 years, it would be inappropriate and insensitive to make efforts to suppress or abandon a name established by Wallace himself. Moreover, *L. nisa* was used as a valid name by Matsumura (1909) after 1899, thus the condition for reversal of precedence ruled by Article 23.9.1 (ICZN 1999: 27) is not met. In the present article, *Lycaena nisa* Wallace is resurrected as the valid name for this lycaenid, with a list of its synonyms.

## Materials and methods

Type specimens relevant to the study were examined in the Natural History Museum, London (**NHMUK**) and the Taiwan Agricultural Research Institute, Taichung (**TARI**). Additional specimens were collected for comparison from Australia, Hong Kong and Hainan, with vouchers deposited in the Department of Life Science, National Taiwan Normal University, Taipei (**NTNU**).

## Taxonomic account

### Famegana nisa

Taxon classificationAnimaliaLepidopteraLycaenidae

(Wallace, 1866) comb. nov.

0ABA4E9A-99AD-5BB1-9E99-8D73350DB319

Figures 1–3

Lycaena nisa Wallace, 1866: 360. Type locality: “Takaw, Formosa”.
Lycaena alsulus Herrich-Schäffer, 1869: 75. Type locality: Rockhampton and Upolu [Australia]. syn. nov.
Lycaena exilis Lucas, 1889: 159, figs 13–15. Type locality: Cooktown to Bowen [Australia] (preoccupied by Lycaena exilis Boisduval, 1852). syn. nov.
Lycaena lulu Mathew, 1889: 312. Type locality: Tongatabu, [Tonga]. syn. nov.
Lycaena gracilis Miskin, 1890: 37. Type locality: Brisbane to Cooktown [Australia]. syn. nov.
Lycaena exiloides Lucas, 1891: 47. Replacement name for Lycaena exilis Lucas, 1889.syn. nov.
Zizera nisa : Matsumura 1909: 480.
Zizeeria alsulus : Waterhouse and Lyell 1914: 106.
Zizera lulu : Rothschild 1915: 390.
Zizera kalawarus Ribbe 1926: 91; Vane Wright and de Jong 2003: 155. Type locality: Celebes. syn. nov.
Zizera alsulus : Seitz 1927: 926.
Zizera taiwana Sonan, 1938: 254. Type locality: “Inrin, Formosa.” syn. nov.
Zizeeria alsulus eggletoni Corbet 1941: 150; Ek-Amnuay 2012: 589. Type locality: Hong Kong, New territory. syn. nov.
Zizina alsulus taiwana : Shirôzu 1944: 37; Shirôzu 1960: 334; Hsu: 2013: 252.
Famegana alsulus : Eliot 1973: 453; D’Abrera 1977: 361; D’Abrera 1986: 651; Parsons 1999: 460; Braby 2000: 839; Braby 2016: 324.
Famegana alsulus alsulus : Common and Waterhouse 1981: 587.

#### Type material examined.

**Wallace**: The specimen of *Lycaena nisa* retrieved in NHMUK,

***Lectotype*** (here designated) (Figs 1–3).

Taiwan • ♀; “♀. Formosa”; “*L. nisa* Wallace”; “Compare Otis Fab.”; “Moore Coll. 1908-203. Formosa.”; reg. no. 720422; NHMUK.

**Sonan**: Three specimens belonging to the type series of *Zizera taiwana* Sonan, 1938 retrieved in TARI, reg. nos. 37, 40, 45.

***Holotype*** (Figs 4–6)

Taiwan • ♂; “Type [round paper, red characters in red circle]”; “Inrin, 30. X. 1932 Col. J. Sonan”; “*Zizera taiwana* Sonan DET. J. SONAN”; “No. 37”.

***Paratypes***

Taiwan • 1♀ (Allotype); “Allo Type [round paper, orange characters in orange circle]”; “Inrin, 30. X. 1932 Col. J. Sonan”; “*Zizera taiwana* Sonan DET. J. SONAN”; “No. 45” • 1 ♀; “Para Type [round paper, green characters in green circle]”; “Inrin, 30. X. 1932 Col. J. Sonan”; “*Zizera taiwana* Sonan DET. J. SONAN”; “No. 40”

**Corbet**: Two specimens belonging to the type series of *Zizeeria alsulus eggletoni* Corbet, 1941 retrieved in NHMUK with the reg. no. 720438.

***Holotype***

Hong Kong • ♂ (Figs 7–9); “♂ Holotype *Zizeeria alsulus eggletoni* Cbt.”; “Hong Kong District. + New Territory 5. IX. 1914. R. W. Barney”, “Type H T [round label with red edge]”, “Brit. Mus. 1921-312”, “Zizeeria GENITALIA slide No. NSC. l.”

***Paratype***

Hong Kong • ♂; “Hong Kong District. + New Territory 5. IX. 1914 R. W. Barney”, “Type Holo-type [round label with red edge]”, “Brit. Mus. 1921-312.” Note: Although this specimen also bears a “holotype” label, the former bears a label with Corbet’s hand-written characters indicating that it is the true holotype.

#### Additional material examined.

Australia • 2 ♂ 2♀; Queensland, Mt. Stuart; 26 March 2017; Y. F. Hsu and M. Braby leg.; 1♂ 1♀; Queensland, Cairns, 30 March 2017; Y. F. Hsu and M. Braby leg. Hong Kong; 1♂; Yuen Long District, Shek Wu Wai; 14 October 2009; Y. F. Lo and W. L. Hui leg; 1♀; Ching Mun CP, Tai Mo Shan; 500m; 20. October. 2009; W. L. Hui leg; 1♀; New Territory, Ngau Tam Mei; 100m; 17 December 2018; Y. F. Hsu leg. Hainan• 3♂; Dongfang, Donghe, Nanran; 18 April 2010; Y. F. Lo leg.

#### Descriptions.

**Lectotype of *Lycaena nisa***

Female (Figs 1, 2). Forewing length 10.9 mm. Head hairy, brown, with medial white band on frons. Antennae dark brown, segmented with white. Proboscis brown. Labial palpus hairy, porrect; third segment slender, pointed at distal end, white but brown dorsally. Compound eyes smooth. Thorax and abdomen dark brown dorsally, white ventrally. Forewing broad, somewhat elongate, termen slightly convex. Hindwing rounded. Wing uppersides uniformly brown. Wing undersides ground color white tinged with gray, spotless except for presence of submarginal bands consisted of faint narrow bands proximally and a series of faint brown spots distally along termen of both wings; dot in cell CuA_1_ prominent, black. Fringe white.

**Holotype of *Lycaena taiwana***

Male (Figs 4, 5). Forewing length 10.9 mm. Morphology conformed to those of *L. nisa*, except metallic purple patches present on uppersides of both wings proximally and those on hindwings.

**Holotype of *Zizeeria alsulus eggletoni***

Male (Figs 7, 8). Forewing length 11.4 mm. Morphology conformed to those of *L. nisa*, except metallic purple patches present on uppersides of both wings proximally, and those on hindwings; submarginal bands on wing undersides slightly paler than those of *L. nisa* and *L. taiwana*.

#### Distribution.

This species occurs in the Oriental and Australian regions, and western Pacific, including southern China, Taiwan, the Philippines, Thailand, Sulawesi, Australia, the Torres Strait islands, Vanuatu, Fiji, Samoa, and Tonga (Eliot 1973; Braby 2000; Vane Wright and de Jong 2003; Ek-Amnuay 2012).

#### Biology.

Larval hostplants of *Famegana nisa* have been reported to include various legume species, such as *Cajanus acutifolius* (F.Muell. ex Benth.) Maesen, *C. pubescens* (Ewart & Morrison) Maesen, *Desmodium elegans* Candolle, *Flemingia macrophylla* (Willd.) Merr., *Indigofera pratensis* F. Muell., *Galactia tenuiflora* (Klein ex Willdenow) Wight & Arnott, *Phyllodium pulchellum* (Linnaeus) Desvaux., *Tephrosia purpurea* (L.) Pers., *Vigna lanceolata* Benth., *V. radiata* (L.) Wilczek and *V. vexillata* (L.) A. Rich (all Fabaceae) (Bascombe et al. 1999; Braby 2000, 2016). Life histories are illustrated and described in Bascombe et al. (1999). Eggs are laid near flowers, upon which the larvae feed. Facultative mymecophily does occur.

## Discussion

**Figures 10–17.**
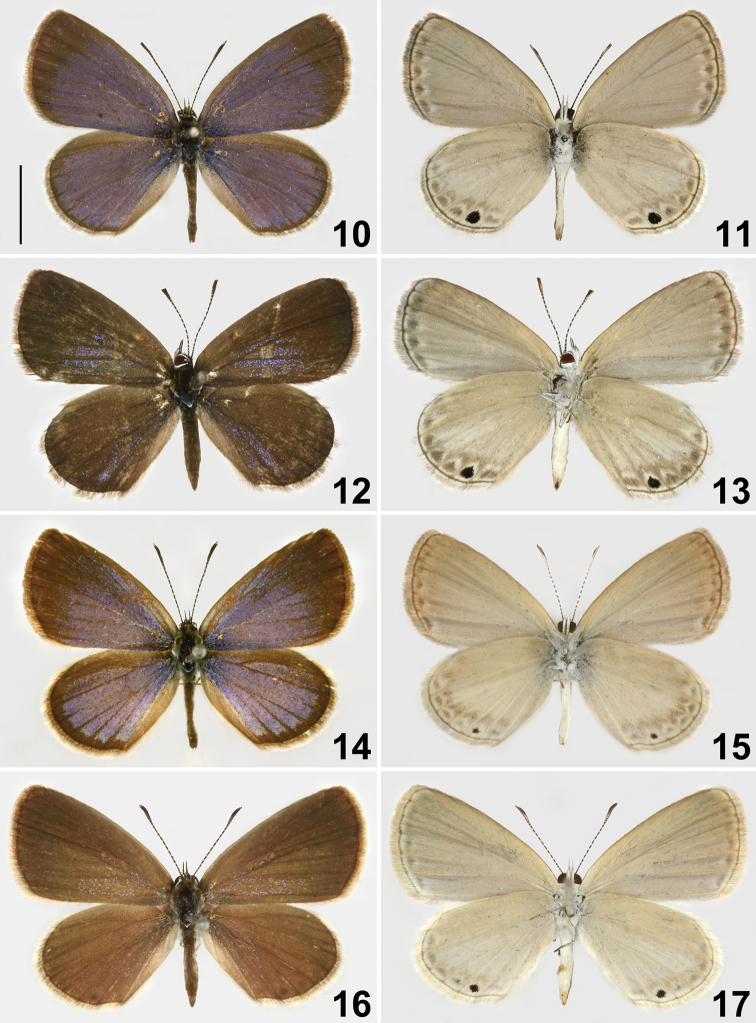
Specimens of *Famegana nisa* (Wallace, 1866) **10, 11** male, Queensland, Cairns **12, 13** female, Queensland, Mt. Stuart **14, 15** male. Hong Kong, Yuen Long District, Shek Wu Wai **16, 17** female, Hong Kong, Ching Mun CP, Tai Mo Shan. Scale bar: 0.5 cm.

*Famegana nisa* dwells on open, grassy habitats, as suggested by its common names ‘Grass Blue’ (e.g., Kimura et al. 2014), ‘Small Grass Blue’ (e.g., Bascombe et al. 1999) or ‘Black-spotted Grass Blue’ (e.g., Braby 2000; Orr and Kitching 2010). This habitat is shared with members of several Polyommatinae, such as *Zizeeria*, *Zizula* or *Zizina*, but *Famegana* can easily be distinguished by its uniformly grayish white undersides of wings, with a single prominent black spot in the cell CuA_1_ of hindwing, and obscure submarginal bands (Common and Waterhouse 1981; Bascombe et al. 1999; Braby 2000; Orr and Kitching 2010). Its male genitalia are also peculiar (Common and Waterhouse 1981), leading Eliot (1973) to establish a monospecific genus for it, stating “unlike those of any other species known to me, the principal peculiarity being the very stout brachia which are hinged wholly to the lateral processes of the tegumen and are capable of only limited movement”. The male genitalia of the species have been illustrated in the literature, including Shirôzu (1960), Eliot (1973), and Bascombe et al. (1999) and are unique in the family Lycaenidae. Although up to four subspecies have been recognized, this species is poorly marked and seasonably variable in wing pattern, and subspecific delimitation is perhaps unnecessary for this species. Specimens in dry season have a reduced black spot, darker ground color on wing undersides, and more distinct submarginal bands on the hindwing undersides (Figs 14–17) when compared to those in wet season (Figs 10–13) according to Bascombe et al. (1999) and Braby (2000).

In addition to the name *Lycaena nisa* of Wallace, another name from Taiwan is available for the species, *Zizera taiwana* Sonan, 1938. Shirôzu (1944) pointed out that *Z. taiwana* is conspecific with *Z. alsulus* based on examination of the male genitalia, but retained *taiwana* as a subspecies of *Z. alsulus*. However, as mentioned above, there is no doubt that *Z. taiwana* represents a junior subjective synonym of *L. nisa*. Moreover, the population from southern China has been assigned to ssp. eggletoni Corbet, 1941, originally described from Hong Kong (Bascombe et al. 1999). As already pointed out by Shirôzu (1944), the specimens from Hong Kong (Figs 14–17), however, are indistinguishable from those from Taiwan (Figs 1–6). Consequently, *eggletoni* Corbet, 1941 should also be regarded as a junior subjective synonym of *L. nisa* Wallace, 1866.

## Supplementary Material

XML Treatment for Famegana nisa
